# Anisakiasis: A Decade of Molecular and Diagnostic Advancements (2015–2026)

**DOI:** 10.3390/ijms27146456

**Published:** 2026-07-20

**Authors:** Juan González-Fernández, Carmen Cuéllar, Alvaro Daschner, Natalie E. Nieuwenhuizen

**Affiliations:** 1 Unidad de Parasitología, Departamento de Microbiología y Parasitología, Facultad de Farmacia, Universidad Complutense de Madrid, 28040 Madrid, Spain; cuellarh@ucm.es; 2 Servicio de Alergia, Instituto de Investigación Sanitaria (IIS), Hospital Universitario de La Princesa, 28006 Madrid, Spain; alvarodaschner@gmail.com; 3 Institute for Hygiene and Microbiology, Julius Maximilian University of Würzburg, Josef-Schneider-Str. 2, 97080 Würzburg, Germany

**Keywords:** *Anisakis*, anisakiasis, allergens, transcriptomics, proteomics, fish, extracellular vesicles, α-Gal syndrome, cancer, diagnostics

## Abstract

Anisakiasis is caused by ingestion of third-stage larvae (L3) of *Anisakis* in fish. The last decade has improved our understanding of *Anisakis* and its clinical manifestations, driven by advancements in high-throughput “omics” techniques and molecular diagnostics. This review summarizes major advancements, focusing on molecular mechanisms underlying its pathogenesis, host–pathogen interactions and novel diagnostic approaches. Taxonomical revisions have refined the *Anisakis* genus, reclassifying several species into *Skrjabinisakis* and *Peritrachelius*. Research has highlighted the critical role of *Anisakis* extracellular vesicles in modulating host immunity. Significant diagnostic breakthroughs include the use of the IgA/IgG4 ratio and antibody avidity profiling to differentiate gastroallergic anisakiasis from chronic urticaria. Identification of α-Gal epitopes in L3 suggests a novel link to α-Gal syndrome and red meat allergy. Ani s 13 and Ani s 14 have been identified as new major allergens, although Ani s 7 remains the gold standard for serological diagnosis. Modern systems biology is revealing how larvae adapt to their host environments, including thermal stress and glucose availability. Finally, emerging evidence suggests potential links between chronic *Anisakis* exposure and pathologies like cancer and sepsis. These advancements underscore the necessity of global clinical awareness and the potential for *Anisakis*-derived molecules as templates for future immunotherapies.

## 1. Introduction

**Figure 1:**
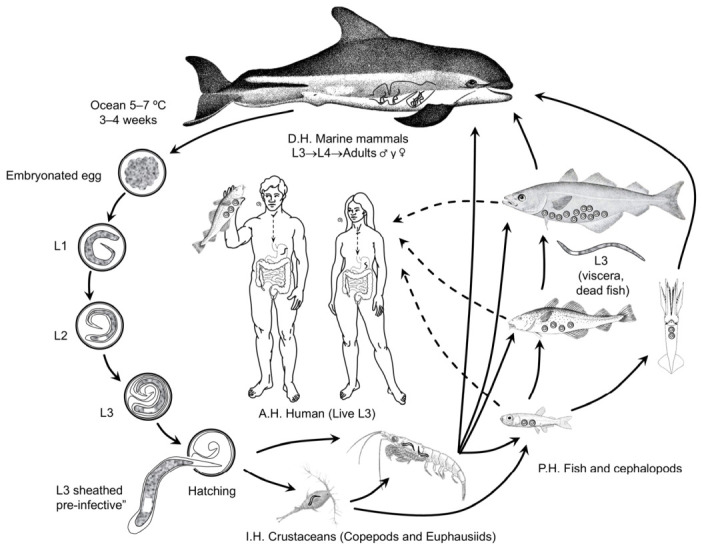
The life cycle of *Anisakis*. D.H.: definitive host, I.H.: intermediate host, A.H.: accidental host, P.H.: paratenic host. Marine mammals, specifically dolphins and whales, serve as the primary definitive hosts for *Anisakis* spp. [3]. The unembryonated eggs of the parasite (~41–58 µm) [4] are excreted into the water via cetacean faeces. Embryonation occurs on the seabed at temperatures between 5–7 °C over a period of 15–22 days [5]. The L1 (first-stage larva) moults to L2 two weeks into embryonation, followed by a second moult to L3 within the egg 3–4 days later [6]. The eggs hatch, releasing pre-infective, swimming L3 larvae (0.33–0.37 mm), which are ingested by tiny crustaceans within the zooplankton, such as copepods and euphausiids. Upon ingestion by copepods, the larvae moult and migrate to the hemocoel, without increasing in size or further developing. In contrast, euphausiids act as obligatory intermediate hosts for *Anisakis*; within them, the L3 grows (4.2–20 mm), moults, and becomes infective to definitive hosts. The cycle is completed when the crustaceans are ingested by definitive hosts or by paratenic hosts such as fish and cephalopods. Within these paratenic hosts, L3 larvae begin to encyst on the surface of visceral organs. Larger fish may prey upon smaller ones carrying L3 larvae, leading to an accumulation of larvae over time. Furthermore, L3 larvae from dead fish or fish eviscerated at sea remain infective for up to six weeks. Upon consuming live L3 larvae in raw or undercooked fish, humans become accidental hosts, and represent a dead end for the parasite life cycle. L3 larvae have also been found infecting other accidental hosts including cats [7] and ducks [8].

*Anisakis* species (spp.) are parasitic marine nematodes whose stage 3 larvae (L3) infect fish as an intermediate host. Cetaceans (whales and dolphins) are the primary host in which larvae moult to stage 4 larvae (L4) and then adult worms, but humans and other land mammals can become accidental hosts by consuming infected raw or undercooked fish and other seafood [1]. The life cycle of *Anisakis* spp. is illustrated in Figure 1. The *Anisakis simplex sensu lato* (*s.l*.) species complex consists of three sibling species that are morphologically indistinguishable during their larval stages: *A. simplex sensu stricto* (*s.s.*). *A. pegreffii* and *A. berlandi* [2]. The species *A. simplex s.s*. and *A. pegreffii* are the most common causes of zoonotic *Anisakis* infection (termed anisakiasis) due to their prevalence in commonly consumed fish. *A.simplex s.s*. is predominantly found in the Arctic–Boreal regions and is the primary cause of anisakiasis in Japan and the North Atlantic, while *A. pegreffii* is the dominant species in the Mediterranean Sea and the Austral Region, accounting for the majority of cases in Southern Europe and parts of East Asia.

In 2021, Safonova et al. [9] suggested revising the *Anisakis* genus to include only five species: *A. simplex sensu stricto*, *A. pegreffii*, *A. berlandi*, *A. ziphidarum* and *A. nascetti*. *Anisakis* is considered a basionym for the physeteris complex (NCBI Taxonomy). Thus, the species *A. brevispiculata*, *A. paggiae*, and *A. physeteris* were placed within the genus *Skrjabinisakis*, whereas *A. typica* was reclassified under the genus *Peritrachelius* [9,10], although some authors still use the genus *Anisakis* [11].

Other parasites within the family Anisakidae of the genera *Pseudoterranova*, *Phocanema* (many of which were formerly named *Pseudoterranova*) [12], and *Contracaecum* are also able to cause infections, although these occur less frequently [13]. Because the definitive hosts of *Anisakis* are whales and dolphins, with wide-reaching migratory ranges, *Anisakis* species are found in virtually every ocean. In contrast, *Phocanema* spp. (whose definitive hosts are pinnipeds such as seals), *Pseudoterranova* spp. (restricted primarily to kogiid whales) and *Contracaecum* spp. (whose definitive hosts include piscivorous birds and pinnipeds) tend to be found localized to coastal areas [12,14]. *Anisakis* larvae are aggressive parasites that burrow deeply into the gastric or intestinal mucosa, leading to acute symptoms (severe abdominal pain, nausea) [15]. Unlike its relatives, *Anisakis* is also notorious for causing allergic reactions, ranging from urticaria to anaphylactic shock.

Anisakiasis can be associated with a variety of symptoms, depending on the infection intensity, host genetics, and prior exposure. This ranges from asymptomatic infection to acute gastrointestinal symptoms to allergic reactions, or a combination referred to as gastroallergic anisakiasis (GAA) [15]. Allergic symptoms seem to require prior infection in the vast majority of cases. This may be attributed to two main factors: first, the mucosal penetration by live larvae facilitates the access of immune cells to allergenic proteins; second, the excretory–secretory (ES) antigens produced by live larvae exhibit greater potency than somatic antigens [16].

Early studies on *Anisakis* and anisakiasis focused on characterization of the disease, morphological studies, speciation of the parasite, and characterization of *Anisakis* allergens. In this narrative review, we summarize the major findings from the last decade of *Anisakis* research, which have been supported by advances in technologies such as next-generation sequencing, proteomics, advanced flow cytometry and new model systems. We will focus on molecular aspects of parasite development and adaptation, diagnostics and host–pathogen interactions but include clinical aspects for a broader perspective.

## 2. Anisakis Transcriptomics, Proteomics and Metabolomics

### 2.1. Transcriptomics

Technologies such as RNA sequencing (RNA-seq) and long-read sequencing enable in-depth analysis of gene expression, alternative splicing and sequence variation, including single-nucleotide variants, within the parasite transcriptome [17,18,19]. In 2016, the first comprehensive transcriptome for *Anisakis* was published. Baird et al. [20] sequenced and annotated the transcriptomes of *A. simplex* s.s. and *A. pegreffi,* the two species responsible for most cases of anisakiasis worldwide due to their prevalence in commercially popular fish. This allowed them to construct an “allergome”, identifying and characterizing novel parasite allergens based on comparisons with known allergen sequence data from other organisms. The next milestone in *Anisakis* research followed in 2019 with the publication of the first draft genome assembly from the Anisakidae family, generated from the L3 stage of *A. simplex sensu lato* (*s.l*), with 7607 predicted coding genes [21]. This provides a reference repository for the Anisakidae family, aiding research into how these parasites function at the molecular level.

Several studies have examined how *Anisakis* spp. respond to alterations in the environment at the transcriptional level. The pathogenicity of *Anisakis* larvae is mainly attributed to their ability to penetrate host tissues by releasing potent bioactive molecules such as proteases from the excretory glands, housed in the pharynx. In 2018, Cavallero et al. [22] performed a comparative transcriptomics analysis of whole larval and pharynges of *A. simplex* and *A. pegreffii* to identify molecules expressed by the pharyngeal tissues that may be linked to pathogenicity. Cysteine-rich secretory proteins (CRISPs) which have previously been identified in flat worms, roundworms, and arthropods were upregulated in the pharynx of both *A. simplex* and *A. pegreffi*. Subsequently, the same research group compared the pharyngeal transcriptome of *Hysterothylacium aduncum*, a non-pathogenic ascaridoid nematode that is common in fish, to that of *A. simplex* s.s. and *A. pegreffi* [23]. In the pharynx of the *Anisakis* spp., transcripts encoding proteolytic enzymes, anaesthetics, anticoagulants and immunomodulatory peptides were upregulated, suggesting that these molecules could contribute to the pathogenicity of the *Anisakis* parasites. In contrast, transcriptomes of non-pathogenic *H. aduncum* larvae and pharynx were enriched in transcripts encoding collagen, peptidases, ribosomal proteins and in heat-shock motifs. This comparative approach highlighted specific molecular adaptations that may contribute to the pathogenicity of *Anisakis* spp. in humans.

While *A. simplex* prefers colder waters, *A. pegreffii* is historically associated with the Mediterranean Sea. The presence of *A. pegreffii* has been increasingly reported over the last 25 years in temperate Atlantic waters along Iberian Peninsula waters [10], suggesting that shifting marine temperatures may influence the global distribution of *Anisakis* species. A recent study performed transcriptomic profiling of *A. simplex* L3 larvae under two temperature conditions: 15 °C (representing the average sea surface temperature in the north-east Atlantic) and 28 °C (simulating extreme ocean warming) [24]. The higher temperature reduced larval lifespan and drove a stress response in the larvae, characterized by upregulation of glycogen metabolism and galactose pathways to mobilize energy reserves. Conversely, the downregulation of cuticle collagen genes and developmental pathways indicated cessation of larval growth. Thus, ocean warming may reshape the distribution of *Anisakis* species and alter transmission dynamics within hosts by shortening the larval lifespan.

To elucidate the molecular mechanisms of larval stage-specific processes and *Anisakis* development, Kim et al. [25] compared the transcriptomes of *A. simplex* L3 and L4 larvae, while Nam et al. [26] compared L3 and L4 transcriptomes for the closely related species *A. pegreffii*. In both species, L3 larvae highly expressed protease-related genes, thought to be crucial for invading host tissues, while L4 expressed collagen synthesis-related genes indicative of the moulting process. Łopieńska-Biernat et al. [21] analyzed how the expression of genes related to carbohydrate metabolism varies across the different life stages (L3 to L5). In L3, the expression profiles indicated a focus on glycogen storage, whereas in L4 and L5, there was a shift towards glycogen catabolism and anabolism of trehalose, a sugar that helps organisms to survive thermal, oxidative, desiccation or osmotic stress and starvation [27]. The increased availability of trehalose could enable the *Anisakis* larvae to adapt to the temperature change of the mammalian host and mature into an adult worm. Most recently, Stryiński et al. [28] analyzed both mRNA and long non-coding RNA (lncRNA) expression in *A. simplex* (s.s.) L3 and L4 collected from the Baltic Sea and northeast Atlantic and found that developmental stage and geographic origin jointly shaped the transcriptomic profile of the parasites. A subset of lncRNAs was linked to regulation of developmentally and population-related genes and targeted pathways such as metabolism, ion transport and protein synthesis, providing insight into the molecular mechanisms of parasite adaptation.

Glucose uptake and metabolism play a critical role in the survival and development of parasitic nematodes and their ability to adapt to the changing environmental conditions of the different hosts [29]. *Anisakis* relies on host-derived glucose to maintain energy metabolism. The intestinal lumen is shrunken in *Anisakis* L3 larvae and only begins to function in L4 larvae [30]. *Anisakis* s.s. L3 and L4 larvae show differences in expression of glucose transporter genes [31]. By comparing the transcriptome of L3 and L4 larvae exposed to glycose compared to controls, Polak et al. [29] identified several differentially regulated pathways related to energy metabolism, translation, cytoskeletal remodelling, and extracellular matrix reorganization. This validates an earlier proteomics study that revealed differences in energy metabolism between *A. simplex* L3 and L4 larvae, in addition to differences in pathways related to oxidative stress, cellular transport, and signalling pathways [32].

Maździarz et al. [17] evaluated the *Anisakis s.s*. transcriptome after exposure to anti-helminthic drugs and found both drug-specific and overlapping transcriptomic changes. Albendazole primarily altered the expression of cuticle-associated genes, ivermectin induced extensive alternative splicing in immune-related pathways, and pyrantel led to widespread single-nucleotide variants in neuronal projection and metabolic genes. Genes involved in detoxification (ABC transporters), oxidative stress (trehalose metabolism), cytoskeletal remodelling, and transcriptional regulation (RNA binding proteins) were affected across treatments. Immune pressure can also affect the transcriptional response. Palomba et al. [33] performed transcriptomic profiling of *A. pegreffii* L3 larvae exposed in vitro to human immature dendritic cells. Upregulated genes were enriched in pathways related to oxidative stress, energy metabolism, and structural maintenance, suggesting metabolic and structural adaption to immune cell-induced stress. Genes involved in cytoskeletal organization and intracellular trafficking were downregulated, which may have reflected developmental arrest of the parasite.

### 2.2. Proteomics

Advances in genome-sequencing technology have led to an increase in protein sequence resources, which has enhanced the potential for proteomics studies relying on mass spectrometry [34]. Over the last decade, *Anisakis* proteomics studies have increased our understanding of the developmental program and adaptive responses of the parasite [29,32,35,36], revealed its use of EVs for immunomodulation [37,38], and identified novel allergens [19,39,40].

Proteomics has been used to characterize the different *Anisakis* species and their larval stages. Arcos et al. [41] compared the proteomes of *A. simplex* s.s., *A. pegreffii* and their hybrid genotype using a quantitative proteomics approach (iTRAQ). They found 37 proteins to be potential discriminant taxonomic biomarkers among *A. simplex*, *A. pegreffii*, and their hybrids. Interestingly, 19 of these differentially expressed proteins, encoded by 10 loci, were *Anisakis* allergens (Ani s 7, Ani s 8, Ani s 12, and Ani s 14). *A. simplex* L3 and L4 developmental stages were compared using tandem mass tag (TMT)-based quantitative proteomics, a powerful mass spectrometry-based technique for the quantification of proteins across multiple samples [32]. The advantages of TMT include reduced variation and high sensitivity. Analysis of the modulated proteins provided the specific proteomic signature of L3 (i.e., pseudocoelomic globin, endochitinase 1, paramyosin) and L4 (i.e., neprilysin-2, glutamate dehydrogenase, aminopeptidase N). The tissue-specific proteome of *A. simplex* s. s. L4 larvae (cuticle, intestine, larval body) was also examined [36].

The secretome (ES products) of *A. simplex* L3 larvae, including EVs, has also been characterized [34,38]. While the total ES fraction contains EVs, the relative abundance of EV-associated proteins is low compared with soluble proteins. Therefore, an enrichment step involving ultracentrifugation is required to reliably profile the EV-associated proteins [38]. Kochanowski et al. [34] detected the allergens Ani s 4, Ani s 2, and Ani s 13 among the ES products, as well as 18 potential allergens, mostly homologs of nematode and arthropod allergens. Based on bioinformatics prediction, 21 proteins were classified into the conventional secretory pathway, and 77 proteins were assigned to unconventional protein secretion. Nine potential pathogenicity-related proteins were predicted, which were predominantly homologs of chaperones, as well as hydrolases, proteases and protease inhibitors. Adduci et al. [38] identified ten of the fourteen recognized *Anisakis* allergens, the most abundant being Ani s 13 and Ani s 1. Apolipophorins (lipid-transport proteins) were the topmost abundant proteins. The authors further characterised N-glycans and the associated glycoproteins present in ES products, finding that pauci-mannose and core-fucosylated N-glycans were major species, while tri-fucosylated and methylated glycans, complex-type, and phosphorylcholine-substituted glycans also occurred.

Studies have also examined changes in the *Anisakis* proteome in response to environmental conditions [29,35,42]. E. coli-derived LPS triggered pathways related to innate immunity, stress response, catalytic activity, oxidation–reduction processes and antioxidant activity in *A. simplex*, indicating that commensal bacteria in the host gut could influence parasite responses [42]. Ivermectin treatment upregulated proteins involved in oxidoreductase activity, immunogenicity, and protein degradation [35], while glucose treatment affected proteins related to ribosome assembly, muscle contraction, and metabolic enzymes [31]. In L3 larvae, the inactive intestine and lack of active glucose transporters may prevent systemic metabolic changes in response to glucose. More proteins were differentially regulated by glucose treatment in the L4 larvae.

### 2.3. Metabolomics

Metabolomics is the identification and quantification of the small-molecule metabolic products (typically < 1500 Da) of a biological specimen at a point in time, providing a snapshot of its physiological state [43]. To date, only one study has evaluated the metabolome of *Anisakis*. Polak et al. [44] analysed the metabolomes of *A. simplex* L3 and L4 by ultra-performance liquid chromatography–mass spectrometry. The results revealed that the developmental stages of *A. simplex* differed in metabolic pathways such as nicotinate and nicotinamide metabolism. L3 larvae mainly activated metabolic pathways for amino acids, starch, and sucrose, while L4 larvae produced prostaglandins and pyridoxine. This indicates that *Anisakis* adapts its metabolism to survive the change from the intermediate fish host to the definitive mammalian host.

While transcriptomic data suggests a genetic shift toward trehalose synthesis and glycogen catabolism in the L4 and L5 stages [21], this metabolomics study detected higher concentrations of trehalose in L3 larvae. This suggests that trehalose plays an important role in the L3 stage, potentially acting as a cryoprotectant or stress response molecule, while the increased capacity for trehalose production observed for the later stages may represent replenishment of trehalose stores.

## 3. Anisakiasis and Allergy

### 3.1. Anisakiasis Pathogenesis

**Figure 2:**
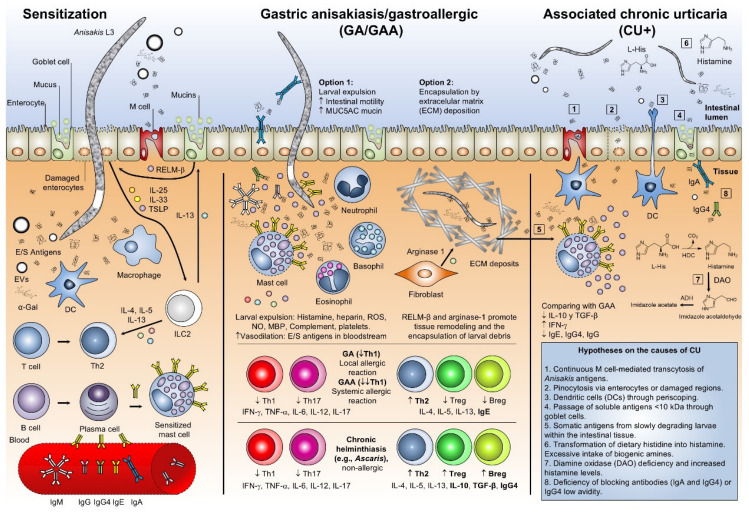
Pathogenesis of *Anisakis* infection and associated allergic reactions. α-Gal: Galactose-α-1,3-galactose (Galα1-3Galβ1-4GlcNAc-R), ADH: Aldehyde dehydrogenase, DC: Dendritic cell, E/S: Excretory/secretory, ECM: Extracellular matrix, EVs: Extracellular vesicles, GA: Gastric anisakiasis, GAA: Gastroallergic anisakiasis, L-His: Histidine, HDC: Histidine decarboxylase, ILC2: Type 2 innate lymphoid cells, L3: Third-stage larvae, LT: T lymphocyte, LB: B lymphocyte, MBP: Eosinophil major basic protein, NO: Nitric oxide, RELM-β: Resistin-like molecule beta, ROS: Reactive oxygen species, TSLP: Thymic stromal lymphopoietin.

Our immune system evolved in coexistence with helminths, which acted as a primary selective force for interleukin genes [45]. Modern sanitary measures have altered this homeostasis by reducing helminth infections, potentially increasing susceptibility to autoimmune and allergic diseases [46], Helminths modulate proinflammatory responses by inducing (or even mimicking) immunoregulatory cytokines (IL-10, TGF-β, activin A) and expanding regulatory T cells (Tregs) and Th2 cells [47,48,49,50]. Beyond soluble antigens, *Anisakis* L3 larvae release extracellular vesicles (EVs) that modulate the host immune response [37,51,52,53]. Furthermore, larval proteases trigger “alarmins” (IL-25, IL-33, TSLP) that stimulate ILC2 cells to produce IL-4, IL-5, and IL-13, promoting Th2 responses and goblet cell differentiation while suppressing Th1/Th17 cells, which are involved in the initial inflammatory response [54] (Figure 2). These effects are strongly influenced by host genetics and epigenetic factors during key developmental stages, as well as the helminth species and infection intensity [55,56,57].

*Anisakis* L3 can penetrate any part of the gastrointestinal mucosa, and infections can be categorized as gastric (GA), intestinal (IA), or ectopic/extraintestinal (EA) anisakiasis [58]. Many cases remain asymptomatic and are detected only during routine screenings [59,60,61,62,63,64,65,66,67,68,69]. This may be dependent on host genetics or reflect a first contact with live larvae in a non-sensitized host; however, some studies identify asymptomatic patients with significant IgE titres, suggesting a state of tolerance [70]. The presence of wider or longer larvae observed in some individuals (asymptomatic and symptomatic) may indicate failure of the immune system to control parasite growth [63,71]. New cases of infection with multiple larvae have also been reported [72]. In symptomatic cases, *Anisakis* can cause severe complications, including acute pancreatitis, intussusception, and intestinal obstructions requiring resection [60,71,73,74,75,76,77,78]. Some authors have remarked that extraintestinal anisakiasis can cause inflammation leading to intraabdominal adhesions in patients with no history of abdominal surgery [77]. Rare ectopic manifestations of anisakiasis have been reported in the mesentery, scrotum, and even the pericardium [79,80]. Such cases highlight the important role of a careful clinical history regarding raw fish intake, which will only be implemented if there is global awareness of *Anisakis* and its clinical implications.

### 3.2. Association with Allergy

Over the last decade, cases of GAA have continued to be reported, particularly in Spain, Italy, and Japan [16,81,82]. Hamada et al. [16] analyzed 42 patients with *Anisakis*-related anaphylaxis and found that all had eaten raw seafood before their first anaphylaxis episode. Patients were followed up for at least one year, and only one patient had two mild hypersensitivity reactions after consuming cooked or processed seafood, suggesting that elimination of cooked or processed seafood is unnecessary for most patients.

Although cases of occupational allergy have been reported [83,84,85,86], in a recent study, the highest risk associated with *Anisakis* sensitization among Croatian fish-processing workers was fishing in the free time and eating raw fish, rather than any attributes related to occupational exposure [85]. An occupational allergy study from Greenland demonstrated that *Anisakis* sensitization levels were high (32.6%), but there was no high risk of being sensitized to *A. simplex* in having ever worked with fish; therefore, sensitization could not be attributed to occupational exposure to *Anisakis* allergens by contact or inhalation [84]. However, occupational allergies associated with *Anisakis* sensitization have been reported from South Africa, where the culinary preference is for well-cooked fish, and no anisakiasis cases have been reported [83]. In vivo studies using mice show that repeated exposure to *Anisakis* antigens alone can trigger allergic airway disease [87,88]. Thus, rare and unusual clinical presentations and their assignation to *Anisakis* allergy cannot be ruled out but should not influence practical clinical considerations or institutional legislation.

Studies indicate that the presence of *Anisakis*-specific IgE correlates with culinary habits rather than total fish intake [89,90]. Prevalence levels are lower in regions where fish is typically cooked or deep-frozen prior to consumption, killing the larvae [91,92]. Furthermore, regulations aiming to prevent cases of anisakiasis such as the Royal Decree 1420/2006 in Spain (that requires 24 h deep-freezing of fishery products intended to be consumed raw or partially cooked) appear to have reduced the prevalence of *Anisakis* sensitization in Spain [93].

Beyond IgE, serum IgA appears to be a key factor in the pathogenesis of chronic urticaria, independent of *Anisakis* sensitization (Figure 2). Research comparing patients with GAA to those with chronic urticaria plus *Anisakis* sensitization (CU+) revealed that CU+ individuals had elevated IgA levels and a higher IgA/IgG4 ratio against major allergens (ES, Ani s 1, 7, and 13) [94]. Additionally, levels of the pro-inflammatory cytokines IL-17A and IFN-γ were lower in CU+ patients. Diamine oxidase (DAO), which degrades histamine in the intestine, may also play a role in CU [95]. Impaired histamine degradation can lead to generalised symptoms including urticarial rash. DAO levels were lower in CU patients without *Anisakis* sensitization (CU−) versus CU+ patients. Thus, while histamine intolerance from the ingestion of histidine-rich fish may drive symptoms in CU− patients, *Anisakis* sensitization may be the primary driver in the CU+ group, independent of DAO activity.

Recently, it was found that *A. simplex* L3 larvae express α-Gal epitopes [96,97], representing a novel sensitization route for α-Gal syndrome (AGS) beyond tick bites [98]. *Anisakis*-sensitized patients (CU+/GAA) have elevated anti-α-Gal IgG, IgE, and IgG4 levels compared to non-sensitized controls and altered IgM/IgG4 profiles, indicating parasite modulation of humoral response [97]. Inhibition assays have confirmed cross-reactivity, with *Anisakis* extracts reducing anti-α-Gal IgG binding by up to 33%, suggesting shared antigenic determinants that could drive IgE class-switching and delayed hypersensitivity to red meat. These data position *A. simplex* as a plausible inducer of α-Gal sensitization, potentially contributing to red meat allergy in high-risk regions like Spain, where anisakiasis prevalence overlaps with urticaria/AGS cases. Further studies on glycolipids and cofactors (e.g., tick co-exposure) are warranted to clarify mechanistic links.

## 4. Diagnosis of Anisakiasis and Anisakis Allergy

### 4.1. Clinical Diagnosis

Clinical diagnosis of anisakiasis consists of a detailed anamnesis with history of raw marine fish intake and confirmation by ImmunoCAP in the lab after obtaining a serum sample [99]. The gold standard of treatment is to observe and to extract the *Anisakis* L3 larvae from gastrointestinal mucosa, but this is not usually possible except in Japan, where a total of 215 endoscopic procedures from 2008 to 2018 [41 (19.1%) cases] were carried out to remove *Anisakis* L3 from the mucosa [100]. The skin prick test (SPT) with *Anisakis* extract is informative but does not necessarily indicate infection or allergy. ImmunoCAP with specific anti-*Anisakis* IgE ≥ 0.35 kIU/L is considered as a reference for routinary diagnosis tests in clinical practice. Current endoscopy guidelines list *Anisakis* as a potential cause of pseudo-tumours [101], as the infection can mimic cancer recurrence. Dead *Anisakis* larvae in the extraintestinal tissues can lead to formation of granulomas which are difficult to distinguish from tumours using PET-CT, and there have been multiple reports of pseudo-tumours in the liver and occasionally at other locations [102,103,104,105,106,107]. Thus, it is important for the general practitioner to be aware of food-associated diseases and obtain a clinical history on ingestion of raw or undercooked fish.

### 4.2. Allergens

The identification of *Anisakis* allergens is critical for the development of component-resolved diagnostics (CRD) to improve the specificity of anisakiasis testing. To date, there are 14 officially characterized *Anisakis* allergens (Table 1), although additional potential allergens have been identified by multiple research groups, particularly with recent advances in high-throughput proteomics screening, genomics, and transcriptomics [20,32,34,37,39,40,108,109,110,111,112,113]. Thorough characterization of these putative allergens and their clinical relevance will be important for improving diagnostics [114].

No new allergens have been officially recognized since 2015, following a decade of intensive allergen characterization. In order to construct an “allergome”, Baird et al. [20] characterized the transcriptome of *A. simplex* and *A. pegreffi* and then used comparative analysis with public databases to identify 36 potential allergens in *A. simplex* and 29 in *A. pegreffii*). These potential novel allergens included cyclophilins and ABA-1 domain-containing proteins. Computational or experimental methods have been used to predict epitopes and 3D structures of some allergens [135,139,140,141]. As one of the last new allergens identified, Ani s 13 (haemoglobin) was further characterized [135,136]. Native Ani s 13 strongly outperformed its recombinant counterpart in diagnostic ELISAs. While the native allergen was detected by 72.1% of sensitized patients (rising to 90% in those with GAA) the recombinant protein was recognized by only 21% of the GAA cohort [136]. This suggests that recombinant allergens should be used with caution in diagnostic tests, as the absence of native glycosylation can significantly alter IgE recognition patterns. The study also demonstrated that *Anisakis* haemoglobin is found mainly as tetramers (43.8%) and octamers (35.7%), the latter accounting for 64.1% of the total heme absorbance. *Anisakis* haemoglobin was further found to be highly immunogenic in rats [142].

*Pseudoterranova decipiens* and *Contracaecum osculatum* are nematodes in the Anisakidae family, closely related to *Anisakis* spp. Homologues of Ani s 2, 3, 5, 7, 8 and 9 and 13 have been detected in *P. decipiens* [39,40,143]. Ani s 2, Ani s 5 and Ani s 13 homologues were identified in *C. osculatum,* as well as a homologue of Asc l 3, an allergen (tropomyosin) from the human roundworm *Ascaris lumbricoides*. This indicates that not only *A. simplex* but also *P. decipiens* and *C. osculatum* should be considered as a potential source of allergic reactions in humans [39,40].

### 4.3. Advances in Diagnosis

Molecular tools for diagnosing anisakiasis and *Anisakis* allergy in patients and detecting *Anisakis* spp. in fish are continuously being updated. The indirect ELISA measuring specific IgE against Ani s 7 is the gold standard for serological diagnosis of anisakiasis [144], since the skin prick test (SPT) and ImmunoCAP are not highly specific [129]. The Trisakis 170 ELISA kit using Ani s 1 and Ani s 7 is used to differentiate patients with GAA and CU [120] and assess allergy severity [145]. New diagnosis tools have been proposed, such as real-time PCR, targeting both the ribosomal ITS region and the mitochondrial COX2 (Cytochrome c Oxidase subunit II) gene [146,147,148], basophil activation test (BAT) [149] or EXiLE [150] methods, but in practice, ImmunoCAP and ELISA continue to lead etiological diagnosis in laboratories. *Anisakis* tropomyosin (Ani s 3) is cause of cross-reactions and lack of specificity because it is a panallergen shared by many organisms from different species. Tropomyosin from other invertebrates, such as shellfish, other nematodes, or dust mites, is a major allergen and cross-reacts with Ani s 3, which is not usually the sensitizing allergen in patients with anisakiasis [149]. In fact, the presence of antibodies against Ani s 3 could be useful to discriminate between patients with and without urticaria [151].

Proteomics has been used in approaches to creating new diagnostic tools. Wang et al. [19] used immunoproteomics to identify sensitive antigens for diagnosis of *A. pegreffi* infection: AniS13 (Ani s 13), Ani609 (VDK51609) and Ani941 (VDK75941). They also developed an indirect ELISA and a luciferase immunoprecipitation assay based on recombinant proteins and used it to detect *A. pegreffi* infection in rats as early as 1 week post infection, using serum. While cross-reactivity to related nematodes infecting humans (e.g., *Ascaris lumbricoides*) was not tested, there was no cross-reactivity to *Schistosoma japonicum*, a more distantly related helminth.

Anisakiasis may often be misdiagnosed due to overlapping symptoms with common gastrointestinal disorders. MALDI-TOF MS biotyping has been adopted in routine clinical practice for the recognition of fungal and bacterial strains due to its ease of use, speed, cost-effectiveness and accuracy, but few studies have applied the technique to clinical parasitology. Thus, Marzano et al. [112] tested MALD-TOF MS profiling of the *Anisakis* proteome to construct the first spectral library for the diagnosis of *Anisakis* infection. The proposed method would work for the identification of extracted larvae. However, the authors highlighted that establishing a diagnostic pipeline would require collection of more *Anisakis* larvae samples from various locations and control samples from other nematodes. Mass spectrometry approaches could also be useful for detecting *Anisakis* proteins in fish samples [111]. A 2 h pipeline for detecting thermostable *Anisakis* allergens using parallel reaction monitoring (PRM)–mass spectrometry was established by Carrera et al. [143].

## 5. Clinical Studies

Human immunology studies on *Anisakis simplex* have revealed a complex network of antibody responses, cytokine patterns, and environmental modulators that shape distinct clinical phenotypes, which include gastrointestinal disease, gastro-allergic anisakiasis (GAA), and chronic urticaria (CU) [152]. Initially, research focused on the immunopathological aspects of disease and how cytokine responses and *Anisakis*-specific IgE, IgG4 and IgA responses to whole larvae antigens and defined allergens (Ani s 1, Ani s 3, Ani s 7, Ani s 13) differ between these phenotypes. Preliminary evidence suggested that the immune response is further influenced by concomitant infections. For example, *Toxoplasma gondii*, which promotes T helper 1 (Th1) responses, characterized by IFN-γ production, modulated the *Anisakis*-driven allergic response in patients with CU [153]. This was the foundation for current research into how polymicrobial infections collectively shape urticarial phenotypes at the molecular level.

Recent studies have refined our understanding of CU associated with *Anisakis* by showing it is often not caused by *Anisakis* alone but by the interplay between *Anisakis* and other chronic co-infections. For example, *T. gondii* seropositivity associates strongly with *Anisakis* sensitization. In contrast, *H. pylori* infection, which enhances IL-17A production, is linked to CU irrespective of *Anisakis* status [154,155]. Epstein–Barr virus (EBV) infection is nearly ubiquitous but shapes cytokine production, particularly IL-6 [155]. Across multiple co-infections, strong positive correlations between anti-*H. pylori* IgG and IL-17A and between anti-EBV IgG and IL-6 highlight a shared inflammatory signature in patients with CU [155,156,157]. Together, these data suggest that *Anisakis* allergy and related urticarial phenotypes result from the interplay between parasite-specific IgE/IgG4/IgA responses, systemic cytokine imbalances, and the immunomodulatory impact of chronic co-infections [155,158].

In the last decade, serum IgA has emerged as a key isotype in *Anisakis*-associated CU [94]. High IgA/IgG4 ratios against excretory–secretory products, Ani s 1, Ani s 7 and Ani s 13 but not the panallergen tropomyosin Ani s 3 characterize patients with CU compared to GAA and healthy controls. Anti-*Anisakis* IgA levels are elevated even in patients without *Anisakis* IgE (allergic sensitization). Reduced systemic levels of IL-10, IFN-γ and IL 17A in CU together with specific correlations between IgA or IgG4 and pro-inflammatory cytokine ratios indicate that IgA participates in the pro-inflammatory response rather than acting solely as a regulatory mucosal antibody [159,160,161,162]. Dietary exposure to inactivated *Anisakis* larvae and altered mucosal permeability may drive polyreactive or inflammation-associated IgA responses that contribute to chronic skin manifestations [94].

Recent studies have refined our understanding of the transition from GAA to CU (Figure 2). IgA (especially anti-*Anisakis* IgA) is elevated in patients with chronic urticaria, and the IgA/IgG4 ratio is associated with CU independently of *Anisakis*-specific IgE [94]. Furthermore, antibody avidity profiling (measuring the binding strength of antibodies) has emerged as a useful biomarker for discriminating between acute and chronic *Anisakis*-related allergic phenotypes. In patients with GAA (the acute stage), anti-*Anisakis* IgE antibodies typically show low avidity, while IgG4 antibodies typically show high avidity. In contrast, in CU (the chronic stage) anti-*Anisakis* IgE antibodies show high avidity while IgG4 antibodies show low avidity. These differences in IgE and IgG4 avidity can be harnessed to improve diagnosis and disease staging [147]. It is also notable that *Anisakis* may act as a source of α-Gal (galactose-α-1,3-galactose) epitopes. Rodero et al. [96] demonstrated that *Anisakis* sp. can express α-Gal structures capable of inducing sensitization, potentially contributing to α-Gal syndrome and delayed meat allergy in some individuals. This opens a new link between parasitic exposure and atypical allergic responses beyond the classical IgE-mediated *Anisakis* response.

Recent studies have revealed a complex interplay between anti-*Anisakis* antibodies, lymphocyte homeostasis, and cytokine signalling, particularly in Crohn’s disease (CD) [163,164]. In healthy individuals, the presence of anti *A. simplex* antibodies, especially IgA, is associated with higher circulating IL-7 levels and a marked reduction of multiple lymphocyte subsets, including CD3^+^αβ^+^, NKT cells, and γδ T cells, suggesting that IL-7 overproduction acts as a compensatory response to antibody-associated lymphopenia [165]. In patients with CD, anti-*Anisakis* IgG and IgM levels (and in some cases IgA) are elevated compared to controls, clustering in patients with ileal involvement and stricturing phenotypes. This positions anti-*Anisakis* antibodies as potential markers of aggressive disease and impaired mucosal barrier function [166].

Immunophenotypic analyses in CD patients has revealed a marked deficit of γδ T cells, particularly CD3^+^CD8^+^γδ^+^ subsets, accompanied by broad reductions in other T cell populations, and these alterations inversely associate with both the presence and levels of anti-*A. simplex* antibodies, implicating γδ T-cell deficiency in increased parasite antigen exposure and in shaping the ensuing humoral response [166]. At the mucosal level, in CD, the tissues display downregulated expression of the common γ chain CD132, upregulated IL-7, and reduced caspase-3, changes that correlate with anti-*Anisakis* IgA and additional isotypes. Collectively, these data indicate that IL-7/IL-7R dysfunction secondary to CD132 deficiency, together with diminished caspase-3-mediated apoptosis, underpins T cell depletion and heightened anti-*Anisakis* seroreactivity in CD, thereby functionally linking defective mucosal immunity to persistent intestinal inflammation and increased vulnerability to this helminth [163].

In recent years, several studies have explored the link between *Anisakis* infection, γδ T cells and sepsis, a life-threatening condition caused by a dysregulated response to infection that can lead to organ failure and death [167,168]. Studies suggest that patients with helminth infections are more susceptible to concurrent microbial infections, including bacterial, protozoan, and viral pathogens [169]. Whether this predisposition stems from parasite immunomodulation, a pre-existing immune deficiency, or both remains unclear. Recently, it has been hypothesized that granulomas induced by helminths may create niches for bacterial proliferation that lead to sepsis, while damage to the mucosal surfaces caused by parasite penetration may allow for spreading of bacteria [170]. Prior *Anisakis* infection is associated with reduced numbers of circulating γδ T cells [168], unconventional T cells that are frequent at mucosal surfaces and play an important role in protective immunity against bacteria. A Spanish study demonstrated an increase in anti-*Anisakis* IgG levels in patients with septic shock compared to healthy control subjects, suggesting a potential link between *Anisakis* infection and the development of sepsis [167]. Patients with sepsis showed an increase in serum anti-*Anisakis* IgG, IgM, and IgA levels 7–10 days after hospital admission [171]. This suggests two possibilities: (1) the potential reactivation of immune memory to *Anisakis* triggered by the sepsis or (2) a recent or ongoing *Anisakis* infection that served as the mechanism for bacterial invasion via the gut. Anti-*Anisakis* IgG was significantly increased in septic shock and correlated positively with inflammatory markers, positive blood cultures, and clinical severity scores, whereas worse sepsis outcomes were associated with reduced anti-*Anisakis* IgA levels and decreased numbers of γδ T cells, which may be indicative of impaired mucosal protection in susceptible patients [167]. Overall, the work suggests that *Anisakis* exposure may affect mucosal immune responses and that, during sepsis, a shift from protective IgA/γδ responses toward IgG-dominated humoral immunity is linked to inflammation, septic shock, and disease severity.

Finally, Napoletano et al. [172] provided direct mechanistic evidence in human monocyte-derived DCs that *A. pegreffii* larvae and their antigens profoundly alter monocyte-derived DC differentiation, maturation, and T cell priming capacity. Their study demonstrated that *A. pegreffii* interfered with DC maturation and signalling through ROS/MAPK pathways, shifting T cell priming away from protective Th1/IFN-γ responses and towards a Th2/Treg profile. This demonstrates that *A. pegreffii* has immunomodulatory activity that can reshape host immune responses to favour its own survival.

Collectively, these human studies support the view that *Anisakis* is not merely a bystander but a frequent, immunologically active co-exposure that unmasks or exacerbates defects in γδ T cell-mediated mucosal surveillance and IL-7-dependent homeostasis, with anti-*Anisakis* antibodies emerging as clinically informative readouts of host–parasite interaction in health and in Crohn’s disease [165].

## 6. Host–Pathogen Interactions

Over the last decade, advances in high-throughput “omics” technologies and 3D models have been increasingly applied to improve our understanding of host–pathogen interactions during helminth infections. Next-generation RNA sequencing technology (RNAseq) has been applied to uncover differentially regulated genes (DEGs) and pathways involved in the response of *Anisakis* larvae to their environment as well as in host responses to parasite infection. Approaches such as cross-talk proteomic analyses are increasingly used to decipher the molecular relationships between *Anisakis* and its hosts [173]. When examining host responses to *Anisakis*, one or more of the following compartments are typically examined: (1) infection with the live larvae; (2) responses to released excretory–secretory (ES) molecules; and (3) responses to somatic antigens derived from the parasite body, which are primarily released upon larval death [51]. ES products have several functions during infection, including penetration of tissues and evasion of host immune responses, but they can also elicit immune responses, and some are allergens [174]. Among the released ES products are extracellular vesicles (EVs), which have been increasingly studied for their role in modulating host responses and the local tissue microenvironment.

### 6.1. Immune Responses to Anisakis in Non-Natural Hosts

The primary hosts of *Anisakis* species are cetaceans, with humans and other land mammals becoming accidental hosts through consumption of infected fish (the intermediate hosts). Murine models have been employed to investigate responses in non-natural hosts [83,175,176]. The response to *Anisakis* in mice is typically a combination of Th2-type immunity to helminth infection and allergic reactions, although the mouse strain affects the responses, echoing the broad spectrum of disease manifestations seen in human hosts [57,83,176,177]. Recent work in rats found that *Anisakis* L3 triggered a strong pro-inflammatory response characterized by intense neutrophil and macrophage infiltration and activation of Th17/IL-17 associated pathways [178,179]. Inflammatory infiltrates and cytokine responses were strongest in the stomach, and tissue-specific microRNA (miRNA) profiles were observed [178].

A 4-week chronic exposure model in mice demonstrated that daily intranasal exposure to *A. pegreffii* CE induced hallmark features of allergic asthma, including airway hyperresponsiveness, eosinophilic inflammation, and structural airway remodelling, driven by Th2 responses [88]. This confirms previous studies that *Anisakis* antigens can independently drive allergic airway disease [87], supporting their potential to cause occupational airway disease [83,86].

Over the last decade, awareness has grown on the impact of the microbiome on immune responses, metabolism and risk of disease. Two studies indicate that *Anisakis* can modulate the gut microbiota. Zeng et al. [180] found that rats infected with *A. pegreffii* showed changes in microbiota diversity, with an increased abundance of pathogenic enteric bacteria, suggesting infection-associated dysbiosis. In silico functional profiling via PICRUSt2 demonstrated that the infected cohort exhibited a significant enrichment in bacterial secretion systems, epithelial cell invasion, chemotaxis, and ABC transporters. Kim et al. [181] demonstrated that ampicillin-induced gut microbiome dysbiosis, characterized by overgrowth of *Proteus vulgaris* and depletion of anaerobes, modified antibody responses to *A. pegreffii* antigens in BALB/c mice. Immunoblotting revealed that several IgG1-reactive spots were gained in the ampicillin-treated group, while multiple IgG2a-reactive spots were diminished or lost, suggesting that gut dysbiosis alters Th1/Th2 polarization.

Recent experimental work has focused on the molecular mechanisms by which *Anisakis* antigens, particularly excretory–secretory (ES) and crude extract (CE) products, manipulate immune cell responses by modulating dendritic cell activation, Toll-like receptor (TLR) signalling, and regulatory T cell (Treg) expansion [177,182,183,184]. Bone marrow-derived DCs (BMDCs) from BALB/c and C57BL/6J mice acquire a semi-mature phenotype after stimulation with *A. simplex* ES or CE, with only modest upregulation of MHC I-II, CD80 and CD86 compared with strong proinflammatory controls such as LPS or CpG [169], suggesting an immature profile typical of helminth-induced DC conditioning. After stimulation, DCs from both mice produced both IL-10 and IL-12, but BALB/c DCs showed significantly higher intracellular IL-12 expression, while C57BL/6J DCs exhibited a higher percentage of IL-10^+^ cells. Functionally, *Anisakis*-conditioned DCs expanded regulatory T cell populations (Tregs) and promoted an immunomodulatory environment, though these effects were highly dependent on host strain and antigen compartment (ES vs. somatic antigens [182]. By antagonizing TLR1/2 and redirecting TLR3/4/7/9 signalling from IL-12 towards IL-10, these antigens effectively curtailed acute Th1-driven inflammation [183]. In contrast, Haryadi et al. [185] observed a significant reduction in Tregs one week following sensitization with *A. typica* extract, suggesting that a loss of regulatory control may allow unchecked Th1/Th2 responses to drive the allergic and inflammatory manifestations of anisakiasis. Collectively, these studies illustrate that while *A. simplex* possesses tolerogenic potential, host genetics and the timing of the immune response likely determine whether the outcome is one of immunomodulation, inflammation or hypersensitivity [177]. This explains the clinical spectrum of anisakiasis, from acute gastro-allergic manifestations to more protracted or subclinical courses and suggests that specific *Anisakis*-derived molecules could be explored as templates for novel immunoregulatory or anti-inflammatory interventions.

Along these lines, a study evaluated the impact of *A. simplex* larval antigens on experimental autoimmune encephalomyelitis (EAE) induced with MOG_35–55_ peptide in C57BL/6J mice [186]. Although antigen treatment led to earlier onset and higher clinical EAE scores, treated mice showed significantly reduced MOG-specific IgG1 in serum and brain, suggesting suppression of autoreactive antibody responses. Treatment initially increased systemic IL-17A and TGF-β levels, but by day 21, serum and brain homogenates showed decreased levels of pro-inflammatory cytokines (IL-6, TNF-α, IL-17A) and a shift toward Treg/Th2 dominance. Overall, the authors propose that *A. simplex* antigen can exacerbate acute EAE clinically while simultaneously downmodulating CNS inflammation, creating an immunoregulatory milieu that could potentially be exploited for therapeutic approaches in demyelinating disease.

### 6.2. Immune Responses to Anisakis in Fish

Like mammals, fish have adaptive immune systems [187]. During migration through fish tissues, L3 larvae can cause local immune reactions and haemorrhages, with the severity of pathology depending on the infection intensity and on the tissue. Palomba et al. [188] compared expression of genes for parasite molecules involved in tissue migration and survival across various tissues of a fish host (*Micromesistius poutassou*) and found tissue-specific parasite adaptation. Interestingly, fish species show differences in their susceptibility to infection and their ability to respond immunologically to the larvae [189]. *Anisakis* ES products downregulated immune response genes in rainbow trout (*Oncorhynchus mykiss*), suggesting modulation of host responses by the parasite [190]. More recently, it was found that infection of Baltic cod (*Gadus morhua*) liver with *Contracaecum osculatum* (a nematode parasite in the family Anisakidae, closely related to *Anisakis*) differentially regulated genes related to metabolism, immune responses, and growth [191]. Activation of inflammatory immune responses was indicated by the upregulation of genes such as *m17* (IL-6 subfamily cytokine), *rela* (p65 subunit of NF-κB), *jun* and *junbb* (part of the AP-1 complex), *tnfaip3* (activated by the NF-κB pathways to control inflammation), and *cxcr4b* and *ccr9*, associated with leukocyte recruitment. Upregulation of semaphorin genes (*sema3h*, *sema3gb*), *crim1* (a BMP regulator) and *acvr1ba* (activin receptor) may reflect tissue remodelling by the host to create a capsule that walls off the parasite. In another study, transcriptomics analysis of the liver of infected *Coilia nasus* (an anchovy species) demonstrated enriched pathways such as natural killer cell-mediated cytotoxicity, the NOD-like receptor signaling pathway, neutrophil extracellular trap formation, and other immune pathways [192].

Zebrafish (*Danio rerio*) are often used in immunological studies, since they can be genetically manipulated; however, they have not often been used to study host responses to *Anisakis* due to the large size of this parasite in comparison to the fish. However, Haarder et al. [193] found that administering ES products from *Anisakis* to TNBS-treated zebrafish in a colitis model reduced pro-inflammatory responses and increased the fish survival rate, again highlighting the immunomodulatory properties of *Anisakis* ES. In other work, administration of the *Anisakis*-derived antimicrobial peptide anisaxin-25 (A-2S) to carp (*Cyprinus carpio*) demonstrated that in addition to its anti-bacterial effects, it has immunomodulatory effects on cytokine expression in host blood cells [194]. Another example of a novel molecule of *A. simplex* being characterized is ANISERP, a serine proteinase inhibitor [195]. Nematode serpins are thought to play an important role in defences against host proteinases and immune evasion.

### 6.3. Host Transcriptomics and Proteomics

Advances in “omics” technologies over the last decade have revolutionized research into helminth–host interactions. A recent article by D’Amelio et al. [196] comprehensively reviews how “omics” studies have contributed to our knowledge on *Anisakis*–host interactions and highlight that more deposited data on *Anisakis* genomes will be necessary in order to fully characterize such biological processes.

Several studies have examined the host transcriptomic responses to the *Anisakis* parasite. Bušelić et al. [179] conducted transcriptomics analysis of stomach and muscle tissues from Sprague–Dawley rats infected with ten *A. pegreffii* larvae by gastric intubation across five timepoints (6, 10, 18, 24 and 32 h post infection). Rats developed severe inflammatory/haemorrhagic lesions in the stomach and muscle tissues, accompanied by gene signature suggestive of IL-17 signalling pathways. While helminths typically induce Th2 responses, and this has also been observed for mice infected with *Anisakis* [83], in rats, the early stages of *Anisakis* infection triggered a pro-inflammatory response characterized by upregulation of genes for chemokines that recruit neutrophils and macrophages (*Ccl2*, *Ccl3*, *Cxcl1*, *Cxcl2*), alarmins that recruit neutrophils (*S100a8* and *S100a9*) and *Mmp3*, a matrix metalloprotease that could contribute to the observed lesions [179].

In an interesting study, Trumbić et al. [197] performed transcriptomic profiling of larvae successfully migrating through the rat as a model of accidental human infection and compared it to that of larvae infecting a representative paratenic host, European seabass (*Dicentrarchus labrax)*. In the rat, *Anisakis* upregulated ribosome-related genes, cell division, cuticle constituents, oxidative phosphorylation, which may indicate an unsuccessful attempt to moult to the next larval stage. In contrast, in fish, larvae showed alterations to metabolic pathways, perhaps preparing for dormancy by triggering autophagy and longevity pathways.

Cross-talk proteomics, in which proteins expressed by the parasite and the host are simultaneously profiled, has allowed insights into the interplay between *Anisakis* and human host cells. Here, a colorectal adenocarcinoma cell line (Caco-2) modelling the intestinal epithelium was stimulated with whole *A. simplex* (s.s.) larvae, and changes to the proteome of both the host cells and *Anisakis* were measured [173]. The Caco-2 cells were also stimulated with *Anisakis* EVs. Incubation of Caco-2 cells with *Anisakis* EVs resulted in greater change to the proteome than co-culture with live *Anisakis* larvae. In addition, differentially regulated proteins were identified in *A. simplex* larvae affected by co-culture with Caco-2 cells, indicating that the parasite adapts its expression of metabolic and structural proteins in response to host signals. However, no in vitro metabolomic studies of *Anisakis* exist.

### 6.4. Human Cell Infection Models

The human colorectal adenocarcinoma cell line Caco-2 is typically used to model the human intestinal epithelium for studying host cell interactions with *Anisakis* [51,173,198]. When grown to confluence, Caco-2 cells differentiate into a two-dimensional (2D) monolayer that mimics the morphology and function of the human small intestine, including the formation of microvilli and tight junctions [51]. This enables studies examining the interactions of *Anisakis* with a model of the human gut wall. However, Caco-2 cell monolayers do not produce mucus and are therefore a limited representation of the intestinal environment [199]. In their review of in vitro and in vivo model systems for anisakiasis, Cavallero et al. [200] argue that simple 2D models often fail to capture the 3D reality of parasite–host interaction, suggesting a move towards organoids. Over the past decade, the integration of organoid technology into infection studies has rapidly expanded, bridging the gap between simple 2D cultures and the complex human system. Organoids are multicellular structures (usually 3D) that recapitulate the physiologic functions of the organ/tissue of origin [52,201]. The establishment of intestinal organoids has greatly improved the ability to study interactions of pathogens with the host gastrointestinal epithelium in vitro [202]. Organoids recapitulate many physicochemical and cellular characteristics of the human host niche that may promote infection, and they allow real-time visualisation of invasion dynamics, which cannot be investigated using animal models. A recent review by White et al. [202] discusses the pros and cons of various 2D and 3D organoids and organ-on-a-chip models for studying particular aspects of infection with gastrointestinal pathogens. Organoids provide a sophisticated in vitro model for investigating the mechanical and chemical interactions between *Anisakis* larvae and the human gastrointestinal mucosa. To date, only one study has used intestinal organs to examine *Anisakis* infection, which examined the effect of extracellular vesicles (EVs) on intestinal cell genes.

### 6.5. Extracellular Vesicles

One of the biggest developments in host–pathogen research over the last decade has been the surge in studies on EVs and how they shape cross-species communication [13]. This has included research on the characterization of *Anisakis*-derived extracellular vesicles (EVs) and their effects on host cells. Extracellular vesicles are small membrane-bound particles (30–1000 nm) released by cells, which carry cargo consisting of proteins, RNA, DNA and other bioactive molecules [203]. Many species use extracellular vesicles for intercellular, cross-phylum communication and host manipulation, including protozoan parasites [204], fungi [205] and helminth parasites [206] including *Anisakis* species [173]. “EVs” serves as a general term for all secreted vesicles with a lipid bilayer; EVs can be broadly categorised three main populations. (1) Microvesicles (ectosomes) range from 100–1000 nm in diameter and are produced through outward budding of the plasma membrane. (2) Exosomes (30–130 nm in diameter) are derived from inward budding of multi-vesicular bodies which subsequently fuse with the cell surface to release their internal cargo [204]. (3) Apoptotic bodies (500–5000 nm) are formed during programmed cell death, when the dying cell fragments into membrane-bound vesicles [207]. EVs can interact with cells in three major ways: (1) receptor-mediated binding, (2) membrane fusion and non-specific entry through the endocytic pathway, and (3) fusion with endosomal membranes. As exosomes are formed through the invagination of endosomal membranes, they display cell surface lipids and proteins on their exterior surface [204]. The transfer of EV cargo to adjacent and even distant cells can have profound effects.

The Minimal Information for Studies of Extracellular Vesicles Guidelines 2023 (MISEV2023) [208] contain information on the latest available approaches to EV isolation and their advantages and limitations for production, separation, and characterisation of EVs from multiple sources, aiming to address EV nomenclature, characterisation, and separation from non-vesicular extracellular particles. In 2023, the helminth research community published a roadmap to help researchers bridge the gap between the MISEV guidelines and the unique challenges of working with parasites, such as limited starting material for studies, definition of pan-helminth EV markers, and identification of rigorous methodologies for in vitro and in vivo studies [209].

Various studies have measured *Anisakis* EVs ranging from 65–295 nm, indicating the presence of both exosomes and microvesicles [37,52,210]. Boysen et al. [210] tracked *Anisakis* EVs by feeding the parasite larvae with a fluorescent lipid analogues, which were incorporated into the EVs. These labelled EVs were internalized by human Thp-1 macrophages, demonstrating that parasite molecules enter the host cell. *Anisakis* EVs contain a complex cargo of proteins and small non-coding RNAs (sncRNAs) that can manipulate host cell responses. Proteomics analysis of *Anisakis* EVs have identified hundreds of proteins involved in RNA silencing, immunomodulation, stress response (e.g., heat shock proteins), tissue invasion (e.g., protease and metallopeptidases), and EV binding (e.g., annexins, tetraspanins) [34,37,173,210]. In a novel approach, Stryiński et al. [173] used TMT mass-labelling on Caco-2 cells treated with EVs to decipher cross-talk between parasite and host proteins by mass spectrometry, identifying 23 specific proteins in the EVs and showing that they directly regulate host proteins involved in cell adhesion and metabolic pathways.

Apart from proteins, *Anisakis* EVs are rich in microRNAs (miRNAs), which are small non-coding RNAs that can silence human genes [52,53] (Table 2). Some miRNA species identified in *Anisakis* EVs are shared among organisms, such as Ape-lin-4-5p, which is homologous to human miR-125, belonging to a highly conserved microRNA family (miR-125a and miR-125b) that can be found throughout diverse species from nematode to humans [211]. It is a common regulatory miRNA used by other helminths to interfere with the host’s immune machinery and promote tolerance [212,213]. Gene targets include those involved in processes such as inflammation, apoptosis, and cell proliferation and/or differentiation. The regulation of genes associated with colorectal cancer progression in human intestinal organoids by *Anisakis* EVs suggests that they may promote a tumourigenic environment [52]. This suggests the need for additional studies examining the long-term health risks associated with repeated exposure to *Anisakis* infection. *Anisakis* EVs also downregulate expression of pro-inflammatory cytokines (IL-6, IL-8) and increase expression of anti-inflammatory IL-10, suggesting that they can suppress defensive host responses, a common strategy in helminth infection [51,173]. However, the acute inflammatory reaction experienced by some human hosts (gastrointestinal symptoms, eosinophil infiltration, allergic reactions) indicates that in the accidental human host, the attempt of the parasite to establish an anti-inflammatory or tolerogenic state via its EVs fails. Nevertheless, immunomodulatory parasite EVs are considered a promising source of novel molecules for treating chronic inflammatory and autoimmune diseases such as inflammatory bowel disease (IBD), rheumatoid arthritis, or multiple sclerosis [214,215]. Parasite miRNA molecules could be powerful tools for knocking down specific disease-causing inflammatory pathways in a therapeutic setting. Thus, elucidating how the EVs efficiently package and transfer RNA (via proteins such as Argonaute proteins) could lead to novel synthetic delivery vehicles for RNA-based anti-inflammatory drugs.

### 6.6. Anisakis and Cancer

**Figure 3:**
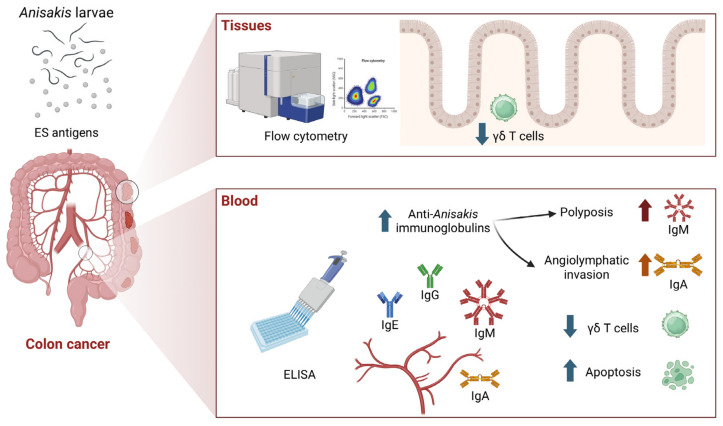
Anti-*Anisakis* antibodies and decreased γδ T cells in patients with colon cancer [226]. There is accumulating evidence that *Anisakis* infections could play a role in carcinogenesis in the gastrointestinal tract by inducing inflammation and modulating immunity.

There has been an increase in studies exploring the link between *Anisakis* (and other parasites) and cancer over the last decade. Case reports suggest that *Anisakis* larvae may have tropism for invading early cancerous mucosa [216,217] or wounds left after surgical resection of digestive system cancers [218]. It is known that certain helminth infections are major contributors to cancer development, such as *Schistosoma* spp. (bladder, liver, colorectal cancers) and *Opisthorchis viverrini* and *Clonorchis sinensis* (cholangiocarcinoma) [219]. Earlier studies suggesting a possible link between *Anisakis* exposure and carcinogenesis [220,221] are supported by recent studies, which have focused mostly on colon cancer (CC) [222,223,224,225]. Patients with CC had elevated levels of anti-*Anisakis* antibodies compared to controls, particularly IgG and IgE [226]. Anti-*Anisakis* IgE levels negatively correlated with γδ T-cell frequencies, especially in patients with CC, and higher IgE positivity was associated with a higher prevalence of γδ T-cell deficit (Figure 3). These findings suggest that *Anisakis* exposure may impair cytotoxic γδ T-cell functions [168], which are crucial for tumour immune surveillance. Apoptosis of both αβ and γδ T-cell subsets was increased in CC, indicating immune exhaustion or suppression, which could facilitate tumour progression and immune evasion [226]. Furthermore, elevated IgA levels have been associated with higher tumour grades and angiolymphatic invasion, markers of aggressive disease [227], suggesting that *Anisakis* exposure may be linked to more malignant tumour phenotypes.

*Anisakis* could contribute to colon carcinogenesis via multiple mechanisms: inducing chronic inflammation, DNA damage, and immune dysregulation. Penetration of *Anisakis* larvae provokes intense mucosal inflammation and may stimulate DNA damage through oxidative stress pathways, potentially leading to mutations and oncogenic transformation. The ability of *Anisakis* to modulate host responses via molecules released in ES products (including EVs) [52] could reduce anti-tumour immunity and create a pro-tumourigenic microenvironment, thereby facilitating cancer progression. In addition, *Anisakis* extract increased cell proliferation and decreased apoptosis in Chinese hamster ovary epithelial cells and altered expression of serum cancer-related miRNAs in rats [225]. Furthermore, *Anisakis*-derived antigens induced inflammation in the intestinal epithelium, further promoting a microenvironment conducive to tumourigenesis [51,224]. Experimental studies employing animal models and in vitro assays are warranted to clarify whether serological and immunological findings conclusively demonstrate a causal relationship between *Anisakis* and cancer. Furthermore, such studies should elucidate whether the parasite actively induces malignant transformation or merely acts as a facilitator in predisposed, immunocompromised individuals.

## 7. Conclusions and Future Directions

The past decade has seen a transition from characterization of clinical disease phenotypes and allergen identification to a focus on better understanding of host–pathogen interactions, immunomodulatory features of *Anisakis,* and its adaptation to the host environment. These studies have identified key factors such as EVs and their protein and miRNA cargo, which facilitate host–parasite communication [52,53,173]. Molecular diagnostics has evolved, with antibody avidity and isotype ratios providing more precise tools to distinguish between GAA and CU phenotypes [94]. The mysterious mechanisms underlying chronic urticaria are also slowly being elucidated [95,154]. Despite these advancements, several critical questions remain, suggesting some avenues for future investigation.

While transcriptomics studies have been performed on *Anisakis* under various conditions, as well as on fish and murine hosts, a significant gap remains in understanding parasite and host responses in human infection. More complex tissue culture models such as organoids may help to address this gap to some extent. The *Anisakis* transcriptome could also be evaluated in larvae recovered from human anisakiasis patients, particularly those with chronic cases, to identify parasite adaptations specific to the human environment.

Preliminary evidence in animal models suggests that *Anisakis* infection may cause intestinal dysbiosis [180,181]. Research is needed to characterize the gut microbiota of GAA patients to determine if specific microbial signatures correlate with the severity of allergic reactions or the transition to chronic urticaria. Understanding the interaction between the parasite, the host immune system and the microbiota could reveal new biomarkers for disease progression. Changes to the microbiome following *Anisakis* infection could also be tracked in relation to the development of cancer, CD, and IBD.

The immunomodulatory properties of *Anisakis*-derived products, particularly EVs, present a frontier for drug discovery. Future research should prioritize evaluating these molecules in murine models of autoimmune diseases, such as IBD and MS. Finally, the emerging link between chronic *Anisakis* exposure and cancer warrants large-scale longitudinal studies. Investigating whether repeated exposure to *Anisakis* can drive or facilitate carcinogenesis remains one of the most pressing questions for the next decade of research.

## Figures and Tables

**Table 1:** Characterized allergens of *Anisakis simplex* in IUIS allergen.org database.

Name	Family	kDa	Sequence	IgE Positivity (%)	References
Ani s 1 ^a^	Kunitz-type serine protease inhibitor; cysteine-rich lustrin-like domain.	21–24	AB100095 Q7Z1K3	85% 88% 93% 61% 62% 67–86% 84%	[115,116] [117] [118] [119] [120] [121] [122]
Ani s 2	Paramyosin	97	AF173004 AAF72796	88%	[123]
Ani s 3	Tropomyosin	41	Y19221 CAB93501	0–13% 46%	[124] [125]
Ani s 4	Cysteine protease inhibitor	9	AM279414 CAK50389	27% 40% 20–41% 35%	[126] [127] [121] [122]
Ani s 5	SXP/RAL-2	15	AB274998 BAF43534	25% 20–26% 35%	[128] [121] [122]
Ani s 6	Serine protease inhibitor	10	AB274999 BAF43535	18%	[128]
Ani s 7	Not determined ^b^	139	EF158010 ABL77410	100% 81% 94% 93%	[129] [130] [119] [120]
Ani s 8	SXP/RAL-2	15	AB300625 BAF75681	25%	[128]
Ani s 9	SXP/RAL-2	14	EU074790 ABV55106	14% 41–43%	[131] [121]
Ani s 10	Not determined ^b^	21	GU187358 ACZ95445	39% 12–49%	[132] [121]
Ani s 11.0101	Not determined ^b^	27	AB555754 BAJ78220	47%	[133]
Ani s 11.0201	Not determined ^b^	16–19	AB555755 BAJ78221	ND 78%	[133] [134]
Ani s 12	Not determined ^b^	31	AB555757 BAJ78223	57%	[133]
Ani s 13	Haemoglobin	37	JX860676.1 AFY98826	64% 72%	[135] [136]
Ani s 14	Not determined ^b^	24–27	LC027371 BAT62430	54%	[137]

^a^ The troponin-like protein of *A. simplex*, also previously named Ani s 1, has had its official nomenclature deprecated, and Ani s 1 now refers to the major allergen, which is a Kunitz-type serine protease inhibitor. The troponin-like protein (GenBank Accession Numbers: AJ012103.2 (gene); CAB58171.1 (protein)) is recognized by 20% of sensitized patients [138]. ^b^ Contains repetitive sequences.

**Table 2:** List of putative gene targets and human homologues of miRNAs identified in *Anisakis* EVs.

miRNA ID	Human Homologue	Potential Human Gene Target(s)	Biological Process	References
Ape-miR-1-3p	miR-1	*MMD*	Immunity and inflammation	[53]
Ape-miR-7-5p	miR-7	*TFF3*, *LOXL2*	Metabolism and healing	[52]
Ape-miR-9-5p	miR-9	*PRTG*	Cell adhesion and motility	[53]
Ape-miR-19	miR-19	*CEP55*, *FAM83C*	Cell cycle and proliferation, MAPK signalling	[52,53]
Ape-miR-27	miR-27	*COL17A1*, *SCD*	Cell adhesion and motility, metabolism and healing	[52]
Ape-miR-57	N/A	*EEF2*	Immunity and inflammation	[52]
Ape-miR-65	N/A	*HSP90AA1*, *RPL27A*, *GPR17*	Cell cycle and proliferation, immunity and inflammation	[52]
Ape-miR-69	N/A	*PGK1*	Angiogenesis	[52]
Ape-miR-71-5p	miR-2 family *	*NAA25*	Cell cycle and proliferation	[53]
Ape-miR-72-5p	miR-124	*MYH9*, *CALR*, *TACC1*, HLA-A	Cell adhesion and motility, antigen presentation	[52]
Ape-miR-97	N/A	*LINGO1*	Signalling	[53]
Ape-miR-100a-5p	miR-100	*TRIB2*	Apoptosis	[53]
Ape-miR-131	N/A	*GPR17*	Immunity and inflammation	[52]
Ape-miR-184	miR-184	*MYH9*, *PABPC1*, *RPL26*	Cell adhesion and motility, mRNA stability, protein synthesis	[52]
Ape-miR-5353-3p	miR-451 family *	*NECAP2*	Intracellular transport	[53]
Ape-miR-5358a-3p	miR-92 family *	*TRIB2*	Apoptosis	[53]
Ape-miR-5364-3p	miR-15 family *	*STARD13*	Cell cycle and proliferation, apoptosis	[53]
Ape-lin-4-5p	miR-125b	*ARID3B*, *EPHB2*, *STARD13*, *BMF*, *FREM1*	TGF-b-PDGF signalling, wound healing, cell cycle and proliferation, cell motility	[52,53]

N/A = not available (no known human homologue). * Family grouping based on seed sequence. miR/miRNA: MicroRNA. Ape: *A. pegreffii.* The miRNAs were identified in *A. pegreffii* EVs.

## Data Availability

No new data were created or analyzed in this study. Data sharing is not applicable to this article.

## Abbreviations

The following abbreviations are used in this manuscript:

AGS	α-Gal syndrome
CC	Colon cancer
CD	Crohn’s disease
CE	Crude extract
CU	Chronic urticaria
CU+	Chronic urticaria with *Anisakis* sensitization
CU-	Chronic urticaria without *Anisakis* sensitization
DAO	Diamine oxidase
EA	Ectopic anisakiasis
EAE	Experimental autoimmune encephalitis
ES	Excretory–secretory
EVs	Extracellular vesicles
GA	Gastric anisakiasis
GAA	Gastro-allergic anisakiasis
IA	Intestinal anisakiasis
IBS	Irritable bowel disease
IFN	Interferon
IL	Interleukin
IU	International units
lncRNA	Long non-coding RNA
miRNA	MicroRNA
MS	Multiple sclerosis
NGS	Next-generation sequencing
RNA-seq	RNA sequencing
TMT	Tandem mass tag
Th	T helper
Treg	Regulatory T cell
TSLP	Thymic stromal lymphopoietin
